# Cognitive behavioral and mindfulness with daily exercise intervention is associated with changes in intestinal microbial taxa and systemic inflammation in patients with Crohn’s disease

**DOI:** 10.1080/19490976.2024.2337269

**Published:** 2024-04-09

**Authors:** Ilan K, Motro Y, Nemirovsky A, Schwartz D, Goren G, Sergienko R, Greenberg D, Slonim-Nevo V, Sarid O, Friger M, Regev S, Odes S, Moran-Gilad J, Monsonego A

**Affiliations:** a The Shraga Segal Department of Microbiology, Immunology, and Genetics, Faculty of Health Sciences, Ben-Gurion University of the Negev, Beer Sheva, Israel; b The National Institute of Biotechnology in the Negev, School of Brain Sciences and Cognition, and Regenerative Medicine and Stem Cell Research Center, Ben-Gurion University of the Negev, Beer-Sheva, Israel; c MAGICAL Group, Department of Health Policy and Management, School of Public Health, Faculty of Health Sciences, Ben‐Gurion University of the Negev, Beer‐Sheva, Israel; d Department of Gastroenterology and Hepatology, Soroka Medical Center, and Faculty of Health Sciences, Ben-Gurion University of the Negev, Beer Sheva, Israel; e Spitzer Department of Social Work, Ben-Gurion University of the Negev, Beer Sheva, Israel; f Department of Health Policy and Management, School of Public Health, Faculty of Health Sciences, Ben-Gurion University of the Negev, Beer Sheva, Israel; g Department of Epidemiology, Biostatistics and Community Health Sciences, Ben-Gurion University of the Negev, Beer Sheva, Israel; h Department of Gastroenterology, Faculty of Health Sciences, Ben-Gurion University of the Negev, Beer Sheva, Israel

**Keywords:** IBD, Crohn’s disease, cytokines, microbiome, psychological intervention, distress

## Abstract

Crohn’s disease (CD) is a chronic inflammatory bowel disease associated with psychological distress and intestinal microbial changes. Here, we examined whether a 3-month period of Cognitive Behavioral and Mindfulness with Daily Exercise (COBMINDEX) intervention, which improves the wellbeing and inflammatory state of CD patients, may also affect their gut microbiome. Gut microbiota, circulating inflammatory markers and hormones were analyzed in 24 CD patients before (T1) and after 3 months of COBMINDEX (T2), and in 25 age- and sex-matched wait-list control patients at the corresponding time-points. Microbiota analysis examined relative taxonomical abundance, alpha and beta diversity, and microbiome correlations with inflammatory and psychological parameters. At T1, CD patients exhibited a characteristic microbial profile mainly constituted of Proteobacteria (17.71%), Firmicutes (65.56%), Actinobacteria (8.46%) and Bacteroidetes (6.24%). Baseline bacterial abundances showed significant correlations with psychological markers of distress and with IFNγ. Following COBMINDEX, no significant changes in alpha and beta diversity were observed between both study groups, though a trend change in beta diversity was noted. Significant changes occurred in the abundance of phyla, families and genera only among the COBMINDEX group. Furthermore, abundance of phyla, families and genera that were altered following COBMNIDEX, significantly correlated with levels of cytokines and psychological parameters. Our results demonstrated that a short-term intervention of COBMINDEX was associated with changes in microbial indices, some of which are linked to psychological manifestations and systemic inflammation in CD patients. Psychological interventions to reduce chronic stress, such as COBMINDEX, appear to be beneficial in mitigating the pathobiology of CD patients, and may thus provide a useful adjunct to pharmacological therapy.

## Introduction

Crohn’s disease (CD), a common form of inflammatory bowel disease (IBD), is a chronic disease characterized by progressive and destructive inflammation of the gastrointestinal tract, the distal small bowel and the large intestine.^1–4^ While the etiology of CD is unknown, its causation is considered to be multifactorial, including immune system imbalance, an altered intestinal microbiome, a genetic predisposition and environmental factors.^4,5^

Previous studies have demonstrated the dysregulated immune system in CD, where disruption of the regulatory cytokine network in the bowel wall is associated with the development and progression of CD.^5–7^ According to these studies, microbial dysbiosis together with a defective gut barrier induce a local immune response, resulting in a pro-inflammatory cytokine loop that overrides anti-inflammatory signals and causes chronic intestinal inflammation.^8^ Inflammatory responses which evolve in CD include pro-inflammatory cytokine (e.g., IL-6, IL-18 and TNFα) release^9^ and an enhanced T helper 17 (Th17) cell response.^10–12^ Cytokines play a key role in the pathogenesis of CD, causing not only gut inflammation but also systemic inflammation which may lead to various extraintestinal manifestations including dysregulated immunity, increased risk for age-related diseases and psychiatric disorders.^13,14^ Regulating the abnormal cytokine pattern seen in CD is the basis for the various biological treatments now in use in CD patients.^15,16^

The gastrointestinal (GI) microbiome has been linked to many diseases, including IBD, where it is frequently proposed as one of the key components affected throughout the disease course.^17,18^ Disease-dependent biodiversity and imbalanced bacterial composition have been associated with CD, where certain commensal bacteria are depleted and the microbial community is less diverse.^19,20^ Decreased representation of several taxa of the Firmicutes phylum, and increased representation of the Gamma-proteobacteria including multiple genera considered potentially pathogenic (e.g., *Escherichia*, *Salmonella*, *Yersinia*, *Helicobacter*, *Vibrio*) were described.^20^ Thus, characterizing the compositional and functional changes in the GI microbiota may lead to specific microbial manipulation as a therapeutic target for CD.^21^

CD patients typically experience negative psychological symptoms,^22^ which may be associated with the onset and progression of IBD.^22^ Psychological distress can be harmful, due to its impact on inflammation via pathways involving the sympathetic nervous system and the hypothalamic-pituitary-adrenal (HPA) axis.^23,24^ The main product of the HPA pathway, cortisol, is secreted in a pulsatile pattern. The pattern of release and the levels of circulating cortisol markedly change in response to environmental and psychological stressors^13,25^ and are linked with a variety of mental health disorders.^13,26^ The possibility that psychosocial interventions may boost immunity and improve immune-related health outcomes has been established in several studies demonstrating that immune system components are influenced by environmental, social, neurocognitive, and behavioral factors.^27–30^ Various treatments are available in clinical practice for reducing negative psychological symptoms (distress, anxiety, and depression) in IBD patients.^22,28,29^ We recently reported a randomized parallel-group physician-blinded trial of cognitive behavioral and mindfulness intervention with daily exercise (COBMINDEX) in adults with mild-to-moderate CD.^31^ This study showed that patients taught COBMINDEX had reduced psychological symptoms compared with the control wait-list group,^31^ and an attenuated disease-associated inflammatory process.^14^

Following our recent report of the significant associations between inflammatory markers and psychological manifestations of CD patients,^14^ in the present study we aimed to determine whether COBMINDEX impacts the gut microbial diversity in accordance with inflammation in patients with CD by examining: 1) the gut microbiota profile of CD patients at baseline and its associations with psychological symptoms and inflammatory markers of disease activity, 2) the impact of COBMINDEX on microbial diversity and whether it associates with changes in psychological symptoms and inflammatory markers.

## Results

### Enrolled Crohn’s disease patients exhibit a characteristic microbial profile which correlates with psychological symptoms and circulating inflammatory markers

**Figure 1.**
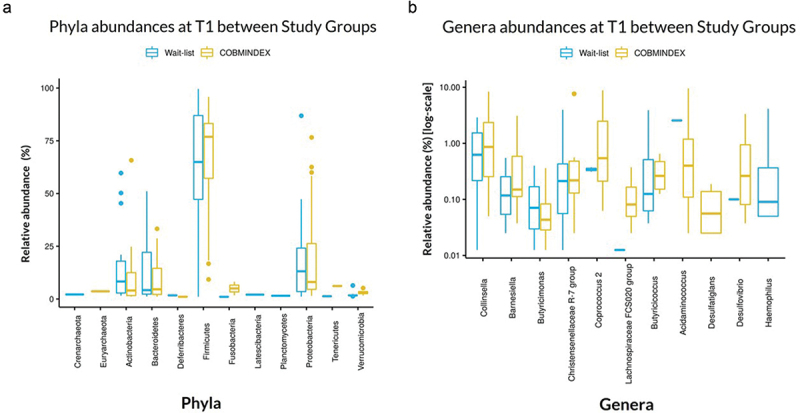
Phyla and genera comparison between wait-list and COBMINDEX at T1 demonstrates no significant differences.

To characterize the gut microbiota profile of CD patients in our cohort, we analyzed stool samples from 49 CD patients at baseline T1 (cohort described in Methods and Table 1). As expected, we found no significant differences in phyla (Figure 1a) and genera (Figure 1b) abundances between the two groups: COBMINDEX (intervention) and wait-list (nonintervention) groups. Notably, the four major phyla, which constitute >98% of the gut microbiome,^32^ are highlighted in our cohort: Firmicutes with a mean abundance of 65.56% (SD = 26.12%, 81% in literature),^17,32^ Proteobacteria with a mean abundance of 17.71% (SD = 23.55%; 5.13% in literature),^17,32^ Actinobacteria with a mean abundance of 8.46% (SD = 13.75%, 3.14% in literature)^17,32^ and Bacteroidetes with a mean abundance of 6.24% (SD = 9.05%, 9.25% in literature)^17,32^ (Table S1).

Plots of phyla (a) and genera (b) abundances between COBMINDEX (*n* = 24) and wait-list (*n* = 25) individuals. LefSe with FDR-adjustment, *p* < .05.

**Table 1.** Demographic characteristics at baseline of Crohn’s disease patients undergoing laboratory studies.

	CD COBMINDEX (*n* = 24)	CD Wait-List (*n* = 25)
Age, mean years (SD)	33.88 (12.97)	32.59 (8.33)
Women, N (%)	18 (72)	15 (56)
Working, N (%)	22 (88)	20 (74)
Current smoking, N (%)	4 (16)	4 (15)
Illness duration, mean years (SD)	5.82 (5.29)	8.67 (8.2)
Harvey-Bradshaw Index:		
Mild disease (5–7), N (%)	11 (44)	10 (37)
Moderate disease (8–16), N (%)	14 (56)	17 (63)
Medications		
Antibiotics, N (%) Steroids, N (%)	1 (4) 1 (4)	0 (0) 2 (7.4)
Immunomodulators, N (%)	3 (12)	7 (26)
Biologics, N (%)	13 (52)	10 (37)

**Figure 2.**
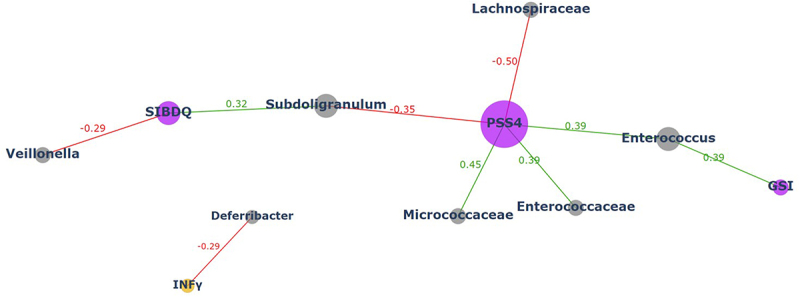
Correlations at T1 demonstrate significant ties between microbial abundance and psychological parameters.

We then sought to determine whether the distinct microbial abundances observed in CD patients are coupled with inflammatory and clinical markers of the disease and/or with psychological manifestations of the disease. As shown in Figure 2 (interactive network) and Table S2, we observed significant correlations between phyla, family and genera abundances and psychological parameters of CD, with no differences between COBMINDEX and wait-list groups (adjusted to age and sex). Notably, most of these significant correlations were between genera and families belonging to *Firmicutes*, known to be decreased in CD, with the main psychological markers of distress (SIBDQ, PSS4, GSI, see Methods section and Figure S1). Among these findings: *Lachnospiraceae* correlated negatively with PSS4 (*r* = −0.37, *p* = .01), *Subdoligranulum* correlated positively with SIBDQ (*r* = 0.35, *p* = .02) and negatively with PSS4 (*r* = −0.351, *p* = .01), and *Enterococcus* correlated positively with GSI (*r* = 0.38, *p* = .01). Furthermore, *Deferribacteraceae* correlated negatively with the pro-inflammatory cytokine INF-γ (*r* = −0.28, *p* = .04). These findings at T1 suggest baseline microbial-psychological-inflammatory relationships among CD patients.

Co-occurrence, interactive networks presenting interactions involving gut microbiome species, psychological parameters and inflammatory markers in both study groups at T1. Each node in the network represents an entity, gray nodes represent bacteria, orange nodes represent inflammatory markers, and purple nodes represent psychological parameters. The size of each node reflects the number of edges associated with that node. The connections (edges) between nodes indicate relationships between the entities; green and red lines (i.e., edges) represent significant positive (*p* < .01) and negative (*p* < .01) correlation between two nodes, respectively.

### COBMINDEX treatment leads to changes in microbial abundance and diversity among Crohn’s disease patients

#### Taxonomic abundance

**Figure 3.**
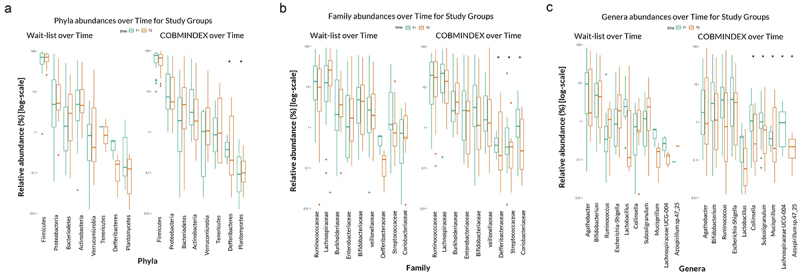
Significant changes in microbial abundance among COBMINDEX and wait-list CD patients.

COBMINDEX was practiced by CD patients for a period of 3 months between T1 and T2; the microbial profile in stool samples was then compared between the two timepoints. Changes in taxonomic abundances among CD patients in both groups, COBMINDEX and wait-list, revealed significant changes in phyla, families and genera only among COBMINDEX (Figure 3a–c, Table S3). For example, an increase in the abundance of the phyla *Deferribacteres* (LDA = 3.51 *p* = .038) (Figure 3a), a decrease in the abundance of the families *Streptococcaceae* (LDA = 3.63 *p* = .038) and *Coriobacteriaceae* (LDA = 3.17 *p* = .043) (Figure 3b), a decreased abundance of genera *Lachnospiraceae* (LDA = 3.08 *p* = .019), and an increased abundance of *Mucispirilum* (LDA = 3.43 *p* = .038) (Figure 3c) occurred only in the COBMINDEX group. These significant alterations, involving changes in inflammatory-related bacteria such as *Streptococcaceae* and *Deferribacteres* (correlated with INFγ at T1), hint at possible improvements in the gut microbiome following COBMINDEX treatment.

Relative abundances of phyla (a), family (b), and genera (c) over time for wait-list (left plots, *n* = 23) and COBMINDEX (right plots, *n* = 23) study groups. Statistically significant changes over time (LefSe with FDR-adjustment, *p* < .05) are highlighted with an asterisk. Bacteria with only one boxplot indicate a 0 relative abundance at one time point.

#### Alpha and beta diversity

**Figure 4.**
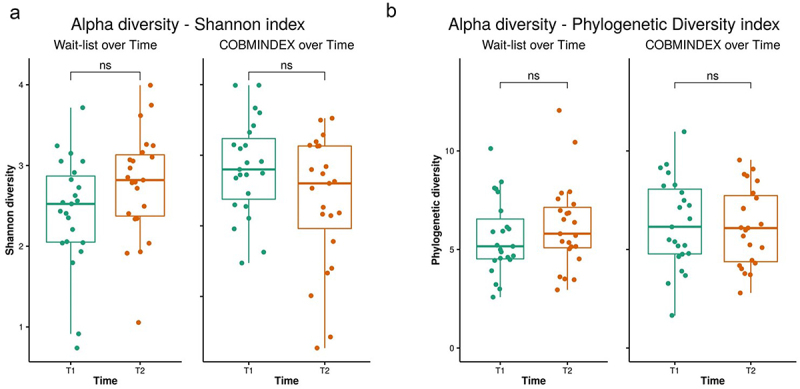
Alpha diversity did not differ in COBMINDEX and wait-list groups over time.

**Figure 5.**
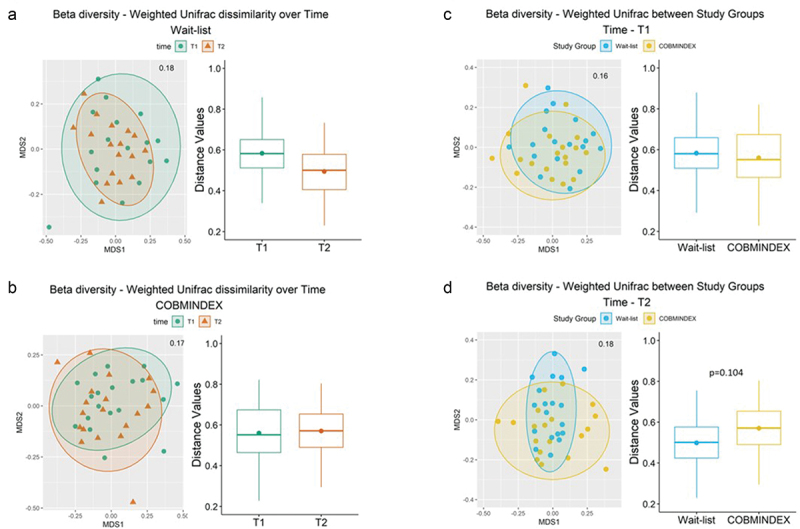
Beta diversity in COBMINDEX and wait-list CD patients over time.

The changes observed between T1 and T2 timepoints following COBMINDEX prompt us to determine whether more global differences in microbiome diversity in the gut can be attributed to COBMINDEX. We found no significant differences in alpha diversity for wait-list and COBMINDEX groups between T1 and T2 time points (Shannon and Faith’s phylogenetic diversity) (Figure 4 and Table S4). In addition, there were no significant differences in beta diversity between T1 and T2 in both wait-list and COBMINDEX groups (Figure 5a–b, Table S5). Of note, although there was no difference between the experimental groups at T1 (Figure 5c), a trend change was observed for the wait-list group at T2 (weighted unifrac index, *p* = .104, Figure 5d), suggesting that further worsening occurred in their gut microbiome as compared with the COBMINDEX group.

Analysis of alpha diversity – Shannon index (a) and Faith’s phylogenetic diversity index (b) – over time for wait-list (left plot, *n* = 23) and COBMINDEX (right plot, *n* = 23) study groups. Paired T-test (FDR-adjusted, *p* < .05).

(a-b) NDMS plot (left) and PERMANOVA (right; 999 permutations, FDR adjusted, *p* < .05) of weighted unifrac dissimilarity index changes over time for wait-list (*n* = 23) and COBMINDEX (*n* = 23) study groups. (c-d) NDMS plot (left) and PERMANOVA (right; 999 permutations, FDR adjusted, *p* < .05) weighted unifrac dissimilarity index between wait-list and COBMINDEX study groups, at T1 and T2.

### Microbial changes following COBMINDEX correlate with psychological and inflammatory markers of CD

**Figure 6.**
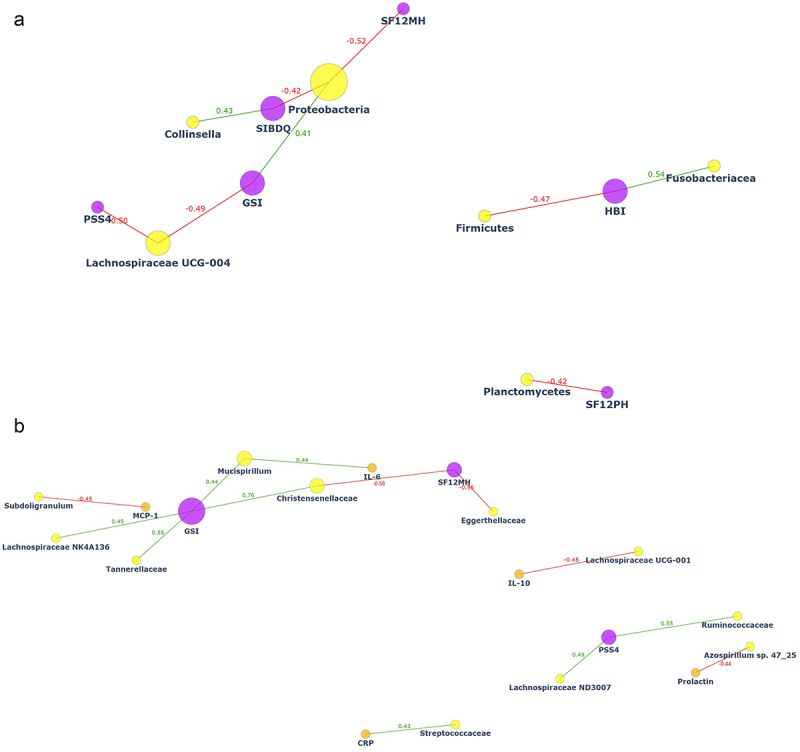
Differential correlations between microbial, psychological, and inflammatory markers in COBMINDEX vs wait-list CD patients.

Since COBMINDEX affected the taxonomic abundance of the gut microbiome, we pursued to analyze whether the microbial changes at T2 are accompanied by changes in inflammatory markers and/or psychological parameters of wellbeing (scores listed in Methods and illustrated in Fig. S1) which we recently observed.^31^ Figure 6a–b (interactive networks) and Table S6A-B highlight the significant correlations between the abundance of microbial phyla, families and genera, with specific inflammatory and psychological parameters among both study groups. Interestingly, abundances of families and genus belonging to *Deferribacteres* and *Firmicutes*, which changed following COBMINDEX (Figure 3), showed correlations with inflammatory markers and/or psychological parameters of distress (Figure 6a, Table S6.A). For example, *Mucispirillum* correlated positively with GSI (r = 0.43, p = .04) and IL-6 (r = 0.44, p = .04), *Streptococcaceae*, a marker of CD relapse that decreased post COBMINDEX, correlated positively with CRP (r = 0.43, p = .04), and *Lachnospiraceae ND3007* correlated positively with PSS4 (r = 0.49, p = .02). All these significant relationships allude to a possible role of *Deferribacteres* and *Firmicutes* in modulating the inflammatory-psychological manifestations of the disease. Notably, when zooming into significant correlations at T2 among wait-list (Figure 6b, Table S6B), *Firmicutes* correlated negatively with HBI (*r*=−.047, p = .01) and its genus *Lachnospiraceae UCG-004*, which showed a significantly decreased abundance among COBMINDEX, correlated negatively with GSI (r = −0.49, p = .01) and PSS4 (r = −0.50, p = .01). Together, these correlations demonstrate an opposite trend among wait-list compared to COBMINDEX and may imply that the effect it has on psychological parameters of distress is at least in part reflected in their gut microbiome.

A. Significant correlations at T2 among COBMINDEX reveal microbial-inflammatory-psychological relationships. Co-occurrence, interactive network presenting interactions involving gut microbiome species, psychological parameters, and inflammatory markers at T2. Each node in the network represents an entity, yellow nodes represent bacteria, orange nodes represent inflammatory markers, and purple nodes represent psychological parameters. The size of each node reflects the number of edges associated with that node. The connections (edges) between nodes indicate relationships between the entities. Green and red lines (i.e., edges) represent significant positive (*p* < .01) and negative (*p* < .01) correlation between two nodes. B. Significant correlations at T2 among wait-list reveal microbial–psychological relationships. Co-occurrence, interactive network presenting interactions involving the gut microbiome species and psychological parameters at T2. Each node in the network represents an entity, yellow nodes represent bacteria, and purple nodes represent psychological parameters. The size of each node is proportional to its degree, reflecting the number of edges associated with that node. The connections (edges) between nodes indicate relationships between the entities. Green and red lines (i.e., edges) represent significant positive (*p* < .01) and negative (*p* < .01) correlation between two nodes, respectively.

## Discussion

The present study evaluated stool and serum samples of CD patients before and after COBMINDEX as compared with wait-list control CD patients. Our results demonstrate a characteristic microbial profile of CD patients consisting of four main phyla. Baseline bacterial abundances showed significant correlations with psychological markers of distress and with IFNγ. Following COBMINDEX, significant taxonomical alterations were revealed among the COBMINDEX group in the abundances of phyla, family, and genera. These abundance changes were accompanied by distinct correlations between psychological parameters and inflammatory markers in COBMINDEX and wait-list controls. Finally, we identified new microbial populations that, based on their associations with inflammatory and/or psychological markers following COBMINDEX, should be further investigated in the context of CD pathobiology.

Previous studies by our group revealed that COBMINDEX improves the quality of life (QoL) of CD patients and that the psychological improvement is accompanied by inflammatory and hormonal changes.^14,31^ These findings allowed us to determine whether better management of psychological distress, at least in part, recovers the microbial changes associated with CD severity. We demonstrated that COBMINDEX is indeed associated with changes in microbial abundance among a variety of phyla, families and genera. The decreased abundances of *Coriobacteriaceae* and *Streptococcaceae* appear to highlight the positive effect of COBMINDEX, as these bacteria are known to increase in CD.^33,34^ Also, a lineage of *Lachnospiraceae*, known to be associated with severe CD,^35,36^ was decreased in COBMINDEX. While the microbial composition did not change among the groups over time, our results did show a trend change in the beta-diversity index among wait-list, but not among COBMINDEX. The very mild differences in the overall microbial composition observed in our cohort, could be best explained by the human microbiome resilience,^37^ the individual’s heterogeneity,^38^ and/or the relatively small sample size, which together with a short treatment time of only 3-months, may mask a more abundant collective group difference. Given these limitations, our findings suggest that COBMINDEX partially recovers the bacterial composition in patients with CD, acting as a key component which can modify the disease process.

Although microbiome alterations in CD may contribute to stress manifestations of the disease, association of psychological markers with microbial signatures was incompletely revealed. We show that both at T1 and T2 time points, families and genera belonging to *Firmicutes* correlated with psychological markers of distress. *Subdoligranulum*, found by Mondot S et al., to be imbalanced among CD patients,^39^ had a positive correlation with the QoL measure SIBDQ and a negative correlation with the psychological stress measure PSS4—both measures were significantly improved post COBMINDEX.^31^ In addition, *Lachnospiraceae*, the most abundant Firmicutes family which is known to be increased in patients with severe CD,^32,35^ correlated positively with PSS4. Notably, *Lachnospiraceae* was found overabundant in CD patients and suggested to contribute to psychological comorbidities via dysregulated bile acids.^36,40^ According to Min Yang.et al., gut dysbiosis leads to disturbed bile acid transformation, impairing the gut barrier and immunity in IBD patients.^41^ Recent research explores the potential role of bile acids as a bridge between the gut microbiome and the brain, suggesting a pathway that could influence the patient’s mental well-being.^42,43^ The decreased abundance of bacteria belonging to *Lachnospiraceae* family in the COBMINDEX group at T2 and their positive correlation with PSS4 and GSI scores highlight *Lachnospiraceae* as an important therapeutic target that should be further explored particularly in the context of impaired bile acid metabolism and psychological distress which contribute to CD pathology.

Our previous research uncovered significant associations between psychological markers of distress with inflammatory and clinical markers of CD.^14^ Accordingly, we sought to find whether microbial–inflammatory relationships explain some of these associations. At T1, *Deferribacteres*, known to have only a small number of genus associated with IBD pathogenesis, showed inverse correlation with IFNγ, and positive correlation with IL-6, two cytokines which can impact the inflammatory and psychological manifestation of CD.^44,45^ Moreover, *Streptococcaceae*, recently found as a microbial marker of CD relapse,^34^ and was in decreased abundance post COBMINDEX, correlated at T2 with CRP – a clinical hallmark of disease progression.^46^ These findings suggest an interesting tie between CD-related bacteria, pro-inflammatory cytokines, and psychological manifestations among CD patients, which should be further elucidated toward identification of disease biomarkers and mechanisms.

We acknowledge several limitations in our study. Firstly, the limitations of cohort size: a larger cohort examined over a longer period with multiple samples of stool, serum, saliva, and/or hair, may better demonstrate the changes in the microbial diversity and abundance, and their associations with inflammatory responses following COBMINDEX in CD patients. Secondly, the use of LEfSe analysis restricted our ability to test interactions between timepoints and groups directly. Moreover, diet is recognized as a risk factor for the development of CD and attributes a pathogenic role to the intestinal dysbiosis inducing an aberrant mucosal immune response.^47^ Therefore, although in our study we did not specify dietary requirements, future research should incorporate dietary modifications to test whether they will enhance the positive effect COBMINDEX had on microbiome.^48,49^ Lastly, future studies may utilize whole-genome metagenomics to explore strain-level characteristics of the gut microbiome in this context.

## Conclusions

Baseline bacterial abundances of CD patients showed significant correlations with psychological markers of distress and with IFNγ. The significant impact of COBMINDEX on psychological symptoms also accompanied alterations in abundances of gut microbiome and systemic inflammation. COBMINDEX may thus be a potential adjunct to the standard of care, that can significantly impact the pathobiology of CD and may underpin a more personalized treatment approach for this patient group.

## Methods

### Cohort

CD patients were recruited from July 2018─July 2020 by advertising at participating hospitals (Soroka Medical Center at Beer Sheva, and Rabin Medical Center at Petah Tikva, Israel) and social media; the study was completed in November 2020. There were no study-related untoward effects. Patients aged 18–75 years with Harvey-Bradshaw Index (HBI) of disease activity in the range 5–16 were eligible. Clinical social workers performed initial screening, and gastroenterologists enrolled patients according to inclusion/exclusion criteria.^31^ Exclusion criteria included: age below 18 or older than 75 years of age, no diagnosis of CD, <1 year of follow-up since diagnosis, change of diagnosis in study period, new medication started in past 3 months, surgery in past 6 months, planned elective surgery, acute surgery during study, pregnancy, planned pregnancy in study period, present/past psychiatric disease/medication, irritable bowel syndrome, not fluent in Hebrew. Irritable bowel syndrome was excluded on clinical grounds (according to Rome 4 criteria).^50^ Patients were then randomized to COBMINDEX or wait-list controls; study physicians and laboratory workers were blinded to patient allocation. Randomization was carried out using the cluster random sampling method,^31^ with proportionate allocation strategy where the fractions were defined by their sex, to COBMINDEX (taught by clinical social workers per standardized protocol on Skype™ over 3 months) or wait-list (control group), as detailed in our recent publication.^51^ The study was carried out between the time points designated as baseline (T1), and study completion after 3 months (T2). Physicians remained blinded to randomization throughout the study. The nature of the study precluded patient participation in protocol-design. All patients remained on medical follow-up, and participation in the study did not affect their treatment. Patients were instructed not to alter their diets or activities in the study period due to its research proven effects on microbiome.^49^ The percentage of smokers in COBMINDEX and wait-list groups was similar. A detailed description of the COBMINDEX protocol can be found in our previously published articles.^14,31,51^

The cohort of patients in the clinical trial was as follows: COBMINDEX 55, wait-list 61; from these patients, we selected a sub-cohort of patients based on who was able to provide both stool and blood samples at time points T1 and T2 for laboratory studies. The sub-cohort included 49 individuals, in two groups: 1) COBMINDEX (*n* = 24, mean age 33.9 years, 72% females), and 2) wait-list (*n* = 25, mean age 32.6 years, 56% females) (Table 1). COBMINDEX and wait-list patients in the laboratory sub-cohort were similar in disease activity and treatments at recruitment (Table 1). Among the COBMINDEX group, one patient took an antibiotic (Ciprofloxacin – 500 mg) for the duration of 14 days during the enlistment process to the study, 21 days prior to beginning the COBMINDEX therapy, therefore we did not consider it as an exclusion criterion. As we previously published COBMINDEX had a significant effect on the wellbeing of CD patients.^31^ The response to COBMINDEX was sorted according to a “Responsiveness Score” detailed in our latest study presenting the effects of COBMINDEX on brain-immune axis regulation.^14^ The patients in the current study, a subgroup of the cohort, are representatives of the responders in the cohort with an average responsiveness score among the COMBINDEX group of 1.26 (std 0.44) compared with −0.07 (std 0.44) among wait-list. Biological samples were provided at T1 and T2 time points. All the samples and data analyzed in this study were collected prior to the onset of the COVID-19 pandemic.

#### Trial registration

Ministry of Health, Israel.

https://my.health.gov.il/CliniTrials/Pages/MOH_2020–02-24_008721.aspx

ClinicalTrials.gov Identifier: NCT05085925.

### Medical and psychological data

At baseline (T1) and after 3 months (T2) we assessed the patients using the following measures^31^ shown in Figure S1:

#### Harvey-Bradshaw Index (HBI)

This questionnaire evaluates disease activity in five questions pertaining to the past day’s symptoms of well-being. Responses are summed to provide HBI. A HBI ≤ 4 indicates disease remission, 5–7 mild disease, 8–16 moderate disease, >16 severe disease.

#### Short Inflammatory Bowel Disease Questionnaire (SIBDQ)

This disease-specific quality of life (QoL) questionnaire relates to the past 2 weeks’ symptoms, general feeling and mood in ten items graded on a 7-point Likert scale (1 = all the time, 7 = never). A higher score indicates better QoL.

#### MOS 12-Item Short Form Survey Instrument (SF-12)

This generic QoL measure has 12 items assessing physical functioning and general health. Scores are summed to yield physical health (SF-12PH) and mental health (SF-12MH) composites, with range 0–100. A higher score indicates better QoL.

#### Brief Symptom Inventory (BSI)

This instrument^52^ measures psychological symptoms in the past month. Its 53 questions assess nine dimensions (depression, somatization, obsession-compulsive, interpersonal sensitivity, anxiety, hostility, phobic anxiety, paranoid ideation, psychoticism) on a 0–4 Likert scale (0 = not affected, 4 = extremely affected). A higher score implies more severe symptoms. The BSI yields a score for each dimension and a summary score called the Global Severity Index (GSI), all with range 0–4.

#### Perceived Stress Scale (PSS-4)

Self-reported questionnaire that measures a person’s evaluation of stressful situations in the previous 1 month of his or her life. The instrument contains 14 statements which measure how unpredictable and overloaded respondents feel their lives are. Respondents rate how often they experience stressful situations on a 5-point Likert scale ranging from ‘never’ (0) to ‘very often’ (4). Higher scores are correlated to more stress.

### Biological data collection

Biological samples (peripheral blood) for measuring inflammation and hormones were drawn from patients at baseline (T1) and after 3 months (T2). Blood samples were taken during morning hours (08:00–13:00) and serum was isolated from Vacuette tube 8 ml with gel (Greiner, Kremsmünster, Austria) and stored in aliquots at −80°C until analysis. The profiling of cytokines/chemokines in serum samples was performed with the CytoFLEX instrument (Beckman Coulter, Brea, CA, USA) using the LEGENDplex™ (BioLegend, San Diego, CA, USA) Human Inflammation Panel 1 (with assay coefficients of variation) [13-plex: IL-1β (13.2%), IFNα2 (23.9%), IFNγ (12.6%), TNF-α (11.1%), MCP-1/CCL2 (8.3%), IL-6 (20.5%), IL-8 (CXCL8–11%), IL-10 (9.1%), IL-12p70 (11.5%), IL-17A (21.4%), IL-18 (7.6%), IL-23 (9.3%), and IL-33 (19.4%)] according to the manufacturer’s instructions. Data were analyzed with the LEGENDplex™ Data Analysis Software Version 8.0. The validity and reliability of the high-sensitivity multiplex assays have been tested in previous studies, overall demonstrating that circulating serum and plasma concentrations of some cytokines correlated accurately with ELISA results.^53,54^ The multiplex assays were found reliable, particularly in the context of smaller studies performed in a small scale and/or when all samples have been collected and can be run at a single time and place.^54^

### Stool sample collection and analysis

DNA from swabs was extracted with the DNeasy PowerSoil kit without modifications (Qiagen, Hilden, Germany). The V4 region of 16S rRNA was amplified using 515F and 806 R primers and processed on a MiSeq instrument per Illumina’s 16S protocol (using a Miseq V2–500 cycle kit to generate 2 × 250 paired-end reads) (Illumina, San Diego, CA, USA).^55^

### Bioinformatics analyses

*Raw Sequence Preparation*: Raw sequence data were imported to the QIIME2 package (v2019.10).^46^ Raw sequence reads underwent primer removal (16S primers from EMP [earth microbiome project], specifically 515F and 807 R for the V4) and DADA2 QC filtering and chimera sequence removal (plugin: dada2 denoise-paired),^56^ with final read counts for 104 analyzed samples ranging from 8467 to 39,647 (average of 14,951).

*Sequence data Analysis*: Representative sequences (output from the DADA2 pipeline) were aligned using the phylogeny plugin (phylogeny align-to-tree-mafft-fasttree)^57,58^ producing a rooted tree, and assigned taxonomic features using the feature-classifier plugin (feature-classifier classify-sklearn)^59^ with the SILVA DB^60^ (v132 at 99%), trained on the 515F and 807 R (V4) subregion.^61^

The amplicon sequence variants (ASVs) feature table, rooted tree, taxonomic assignments and metadata were imported into R (v4)^62^ using the package file2meco (v0.5.0)^63^ for further analyses with the R package microeco (v0.14.1).^64^ The samples were then rarefied at a sampling depth of 8000 for downstream analyses.

### Microbiome and statistical analyses

For T1 samples (*n* = 49), a comparison of relative abundance of phyla, families and genera between COBMINDEX and wait-list samples was performed using the LefSe method^65^ (with FDR adjustment), with p-values < .05 considered significant. Beta diversity (using the indices Bray-Curtis, weighted and unweighted unifrac) was measured between COBMINDEX and wait-list samples, with non-metric multidimensional scaling (NDMS) ordination plots and group significance determined by PERMANOVA test (with 999 permutations and FDR adjustment), with p-values <.05 considered significant. Differential taxa abundance (using the LefSe method at the family taxonomic level) was also measured between COBMINDEX and wait-list samples. Finally, for T1 (*n* = 49) and T2 (*n* = 48) samples (separately), a correlation analysis (Spearman’s correlation) between microbial abundances from the ASVs feature table at the phylum, family and genus taxonomic levels, with inflammatory and psychological markers, was conducted using the cal_cor function from the R package microeco with p-value <.05 considered significant.^66^

For the COBMINDEX (*n* = 23 patients, 46 samples) and wait-list (*n* = 23 patients, 46 samples) groups, samples were analyzed over time (T1 vs. T2) for changes in relative abundance at the phylum, family and genus taxonomic levels using the LefSe method,^67^ where variations were considered significant when the linear discriminant analysis (LDA) score was greater than 3 and/or the p-value from the LefSe test was <0.05. Due to outliers, 1 COBMINDEX and 2 wait-list patients were taken out. Changes over time in alpha diversity (using the indices Shannon, Simpson and phylogenetic diversity [PD]) were tested using the paired T-test (with FDR adjustment), with p-values < .05 considered significant. Changes over time in beta diversity (using the indices Bray-Curtis, weighted and unweighted unifrac) were analyzed using non-metric multidimensional scaling (NDMS) ordination plots and group significance was determined by PERMANOVA test (with 999 permutations and FDR adjustment), with p-values <.05 considered significant.

Co-occurrence networks were generated based on correlation scores. Network visualization and the positioning of the nodes were calculated according to the force directed Fruchterman & Reingold algorithm used for calculating layouts of simple undirected graphs.^68^ The algorithm was implemented using nx.draw function via the ‘pos’ parameter in the ‘NetworkX’ python package v1.11. Node degree was calculated using the nx.degree function. Visualization was generated using ‘Plotly’ python package v4.9.0.

## Supplementary Material

Supplemental Material

supp tables 140324 clean.docx

## Data Availability

The data supporting this study’s findings of this study are available on request from the corresponding author [A.M]. The data are not publicly available due to their containing information that could compromise the privacy of research participants.
