# Supplementary figures and images for: Cognitive behavioral and mindfulness with daily exercise intervention is associated with changes in intestinal microbial taxa and systemic inflammation in patients with Crohn’s disease
Source: Gut Microbes. 2024 Apr 9;16(1):2337269. doi: 10.1080/19490976.2024.2337269 (PMC11005811; doi:10.1080/19490976.2024.2337269)

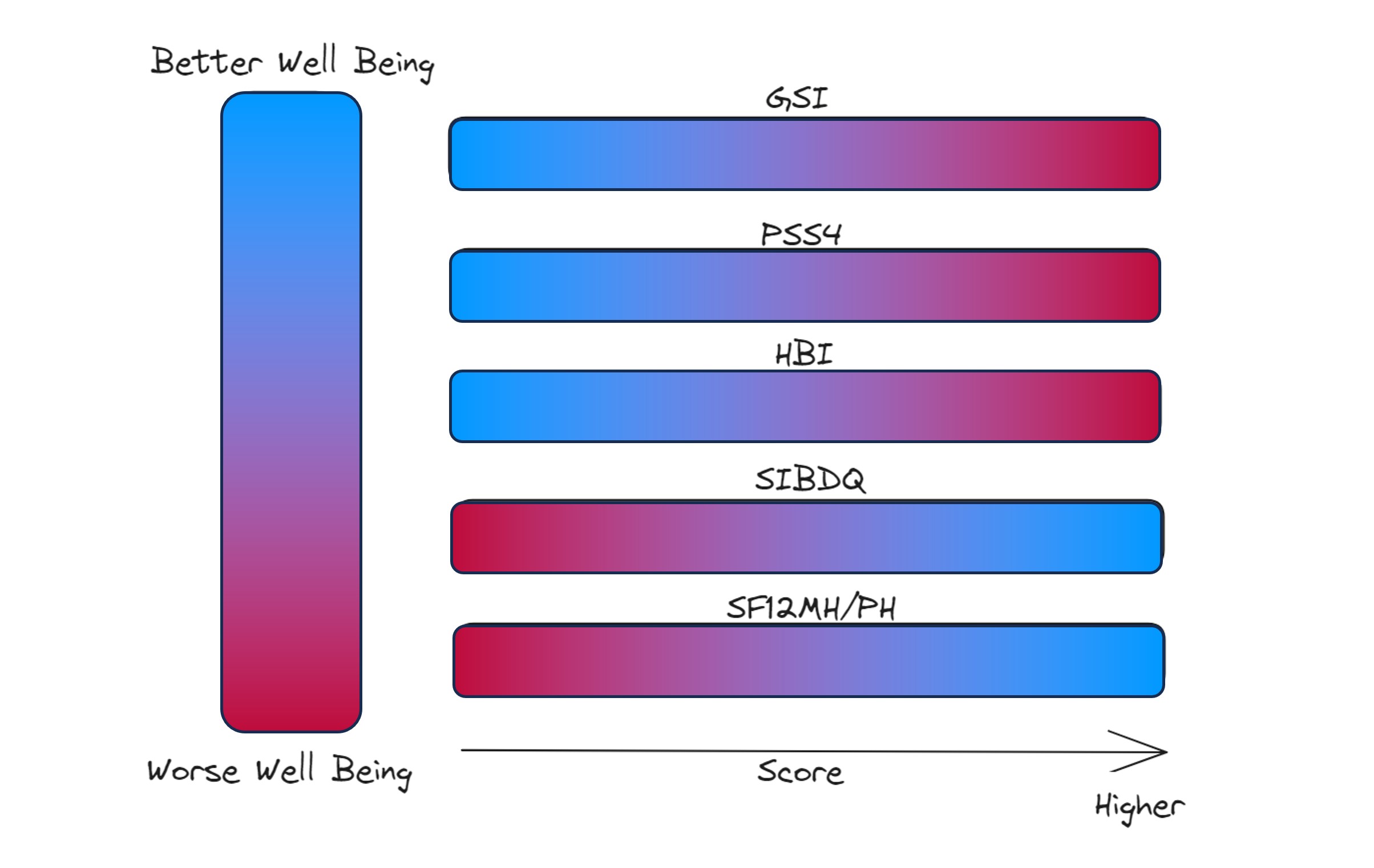

Supplement: Supplemental Material [file KGMI_A_2337269_SM2860.jpg]
