# Supplementary material for: Cognitive behavioral and mindfulness with daily exercise intervention is associated with changes in intestinal microbial taxa and systemic inflammation in patients with Crohn’s disease
Source: Gut Microbes. 2024 Apr 9;16(1):2337269. doi: 10.1080/19490976.2024.2337269 (PMC11005811; doi:10.1080/19490976.2024.2337269)
Supplement: supp tables 140324 clean.docx [file KGMI_A_2337269_SM5978.docx]

| **Wait-list** | | | | | | **COBMINDEX** | | | | | |  |
| --- | --- | --- | --- | --- | --- | --- | --- | --- | --- | --- | --- | --- |
| **IQR** | **max** | **min** | **Median** | **sd** | **Mean** | **IQR** | **Max** | **Min** | **Median** | **sd** | **Mean** | **Phylum** |
| 3.83 | 51.07 | 0 | 1.375 | 12.784 | 7.599 | 7.25 | 33.275 | 0.05 | 2.01 | 9.057 | 6.241 | **Bacteroidetes** |
| 41.59 | 99.58 | 1.062 | 70.1 | 26.848 | 62.646 | 43.13 | 95.837 | 9.275 | 75.95 | 26.194 | 65.562 | **Firmicutes** |
| 0 | 1.75 | 0 | 0 | 0.376 | 0.129 | 0 | 1.062 | 0 | 0 | 0.213 | 0.05 | **Deferribacteres** |
| 19.13 | 86.86 | 0.037 | 6.96 | 21.159 | 16.268 | 35.131 | 76.6 | 0 | 7.38 | 23.555 | 17.713 | **Proteobacteria** |
| 8.7 | 59.7 | 0 | 4.45 | 16.289 | 11.853 | 9.05 | 65.75 | 0 | 1.73 | 13.753 | 8.464 | **Actinobacteria** |
| 0.181 | 6.4 | 0 | 0 | 1.354 | 0.571 | 0.331 | 5.175 | 0 | 0 | 1.474 | 0.828 | **Verrucomicrobia** |
| 0 | 0.912 | 0 | 0 | 0.202 | 0.058 | 0 | 3.65 | 0 | 0 | 0.728 | 0.153 | **Euryarchaeota** |
| 0 | 1.05 | 0 | 0 | 0.233 | 0.077 | 0 | 8.187 | 0 | 0 | 1.660 | 0.397 | **Fusobacteria** |
| 0 | 1.287 | 0 | 0 | 0.257 | 0.052 | 0 | 6.137 | 0 | 0 | 1.226 | 0.251 | **Tenericutes** |
| 0.01 | 1.937 | 0 | 0 | 0.438 | 0.158 | 0 | 0.55 | 0 | 0 | 0.112 | 0.027 | **Planctomycetes** |
| 0 | 2.137 | 0 | 0 | 0.436 | 0.133 | 0 | 0.75 | 0 | 0 | 0.150 | 0.035 | **Crenarchaeota** |
| 0 | 2.05 | 0 | 0 | 0.411 | 0.094 | 0 | 0 | 0 | 0 | 0 | 0 | **Latescibacteria** |

**Supplementary tables:**

**Table S1. Relative taxonomical abundances of phyla among COBMINDEX and wait-list control CD patients at T1.** Relative abundances at phyla taxonomic level among CD patients study groups (COBMINDEX and Wait-list) at T1. No significant changes were observed between the groups (Wilcoxon, FDR p > 0.05).

| **Taxa** | **Bacteria** | **Parameter** | **Correlation** | **P value** |
| --- | --- | --- | --- | --- |
| **Phylum** | Deferribacteres | INFγ | -0.289 | 0.048 |
| **Family** | Micrococcaceae | PSS4 | 0.450 | 0.001 |
|  | Lachnospiraceae | PSS4 | -0.378 | 0.008 |
|  | Enterococcaceae | PSS4 | 0.387 | 0.006 |
|  | Deferribacteraceae | INFγ | -0.289 | 0.048 |
| **Genus** | Veillonella | SIBDQ | -0.293 | 0.042 |
|  | Subdoligranulum | SIBDQ | 0.319 | 0.026 |
|  | Subdoligranulum | PSS4 | -0.351 | 0.014 |
|  | Enterococcus | PSS4 | 0.3873 | 0.006 |
|  | Enterococcus | GSI | 0.386 | 0.006 |

**Table S2. Microbial taxa positively correlate to psychological and inflammatory markers among CD patients at T1.** Significant correlation's (Spearman, p-value < 0.05) between microbial abundance (at the phylum, family and genus taxonomic levels) and psychological and inflammatory markers among CD patients at T1

| **Taxa** | **Bacteria** | **LDA** | **P value (adjusted)** | **Direction of Change** |
| --- | --- | --- | --- | --- |
| **Phyla** | Deferribacteres | 3.512 | 0.038 | Increase |
|  | Planctomycetes | 2.545 | 0.035 | Increase |
| **Family** | Deferribacteraceae | 3.81 | 0.038 | Increase |
|  | Streptococcaceae | 3.63 | 0.038 | Decrease |
|  | Coriobacteriaceae | 3.176 | 0.043 | Decrease |
| **Genera** | Collinsella | 3.685 | 0.043 | Decrease |
|  | Subdoligranulum | 3.469 | 0.015 | Decrease |
|  | Mucispirillum | 3.439 | 0.038 | Increase |
|  | Lachnospiraceae UCG-004 | 3.079 | 0.019 | Decrease |
|  | Azospirillum sp. 47_25 | 2.986 | 0.038 | Decrease |

**Table S3. Microbial abundancy changes are induced by COBMINDEX.** Significant changes (FDR p-value < 0.05) in microbial abundance (at the phylum, family and genus taxonomic levels) among COBMINDEX CD patients between T1 and T2, using the LefSe analysis (Methods).

| **Wait-list** | | | | | | **COBMINDEX** | | | | | |  | |
| --- | --- | --- | --- | --- | --- | --- | --- | --- | --- | --- | --- | --- | --- |
| **IQR** | **max** | **min** | **median** | **sd** | **mean** | **IQR** | **max** | **min** | **median** | **sd** | **mean** | **Measure** | **Time** |
| 0.818 | 3.71 | 0.74 | 2.52 | 0.68 | 2.43 | 0.858 | 3.99 | 1.47 | 2.798 | 0.68 | 2.83 | **Shannon** | **T1** |
| 0.759 | 3.9 | 1.06 | 2.81 | 0.66 | 2.77 | 1.168 | 3.52 | 0.27 | 2.60 | 0.93 | 2.35 | **Shannon** | **T2** |
| 2.02 | 10.11 | 2.58 | 5.16 | 1.9 | 5.62 | 3.28 | 11 | 1.64 | 6.15 | 2.27 | 6.32 | **PD** | **T1** |
| 2.05 | 12.05 | 2.95 | 5.80 | 2.16 | 6.19 | 3.35 | 9.54 | 2.8 | 6.08 | 1.97 | 6.17 | **PD** | **T2** |

**Table S4. COBMINDEX did not affect Alpha diversity among CD patients.** Changes in alpha diversity [Shannon and Faith’s phylogenetic diversity (PD) indices) for both study groups (Wait-list and COBMINDEX] over time. No statistically significant changes were observed (paired T-test, FDR p > 0.05).

| **Groups** | **Time** | **F** | **R2** | **p-value** |
| --- | --- | --- | --- | --- |
| Wait-list | T1 vs T2 | 0.757 | 0.0231 | 0.651 |
| COBMINDEX | T1 vs T2 | 0.809 | 0.0220 | 0.591 |
| Wait-list vs COBMINDEX | T1 | 0.647 | 0.0177 | 0.748 |
| Wait-list vs COBMINDEX | T2 | 1.494 | 0.0400 | 0.104 |

**Table S5. Beta diversity (weighted unifrac) did not significantly change in COBMINDEX and wait-list control groups between T1 and T2 time points.** Changes in beta diversity (weighted unifrac dissimilarity indices) for both study groups (wait-list and COBMINDEX) over time, and between study groups at each time point. No statistically significant changes were observed for both study groups over time, and between the study groups at each time point (weighted unifrac dissimilarity, PERMANOVA, 999 permutations, p < 0.05).

|  | **Bacteria** | **Parameter** | **Correlation** | **P-value** |
| --- | --- | --- | --- | --- |
| **Phylum** | Deferribacteres | IL-6 | 0.443 | 0.044 |
|  | Deferribacteres | GSI | 0.437 | 0.041 |
| **Family** | Streptococcaceae | CRP | 0.434 | 0.048 |
|  | Deferribacteraceae | GSI | 0.437 | 0.041 |
|  | Christensenellaceae | GSI | 0.762 | 0.000 |
|  | Tannerellaceae | GSI | 0.575 | 0.005 |
|  | Christensenellaceae | SF12MH | -0.576 | 0.004 |
|  | Eggerthellaceae | SF12MH | -0.549 | 0.008 |
|  | Ruminococcaceae | PSS4 | 0.551 | 0.007 |
| **Genus** | Subdoligranulum | MCP1 | -0.447 | 0.036 |
|  | Mucispirillum | IL-6 | 0.443 | 0.044 |
|  | Lachnospiraceae UCG-001 | IL-10 | -0.449 | 0.035 |
|  | Azospirillum sp. 47_25 | Prolactin | -0.442 | 0.039 |
|  | Mucispirillum | GSI | 0.437 | 0.041 |
|  | Lachnospiraceae NK4A136 group | GSI | 0.449 | 0.035 |
|  | Lachnospiraceae ND3007 group | PSS4 | 0.492 | 0.019 |

**Table S6A. Microbial abundances post COBMINDEX correlate with psychological and inflammatory markers.** Significant correlations (Spearman, p-value < 0.05) between microbial abundances (at the phylum, family and genus taxonomic levels) and psychological and inflammatory markers among COBMINDEX CD patients at T2.

|  | **Bacteria** | **Parameter** | **Correlation** | **P-value** |
| --- | --- | --- | --- | --- |
| **Phylum** | Planctomycetes | SF12PH | -0.4195658 | 0.036808 |
|  | Firmicutes | HBI | -0.4710962 | 0.017454 |
|  | Proteobacteria | GSI | 0.41393944 | 0.039682 |
|  | Proteobacteria | SIBDQ | -0.4163301 | 0.03844 |
|  | Proteobacteria | SF12MH | -0.5207692 | 0.008403 |
| **Family** | Coriobacteriaceae | SIBDQ | 0.42678327 | 0.033367 |
|  | Fusobacteriaceae | HBI | 0.5431092 | 0.005023 |
| **Genus** | Lachnospiraceae UCG-004 | GSI | -0.4914485 | 0.012598 |
|  | Collinsella | SIBDQ | 0.42678327 | 0.033367 |
|  | Lachnospiraceae UCG-004 | PSS4 | -0.50471 | 0.010081 |

**Table S6B. Microbial abundances among wait-list correlate with psychological markers**. Significant correlations (Spearman, p-value < 0.05) between microbial abundances (at the phylum, family and genus taxonomic levels) and psychological markers among CD wait-list patients at T2.
